# Untargeted Metabolomics Based Prediction of Therapeutic Potential for Apigenin and Chrysin

**DOI:** 10.3390/ijms24044066

**Published:** 2023-02-17

**Authors:** Cole Cochran, Katelyn Martin, Daniel Rafferty, Jennifer Choi, Angela Leontyev, Akanksh Shetty, Sonali Kurup, Prasanth Puthanveetil

**Affiliations:** 1 Chicago College of Osteopathic Medicine, Midwestern University, Downers Grove, IL 60515, USA; 2 Department of Biomedical Sciences, College of Graduate Studies, Midwestern University, Downers Grove, IL 60515, USA; 3 Arizona College of Osteopathic Medicine, Midwestern University, Glendale, AZ 85308, USA; 4 College of Pharmacy, Ferris State University, 220 Ferris Drive, Big Rapids, MI 49301, USA; 5 Department of Pharmacology, College of Graduate Studies, Midwestern University, Downers Grove, IL 60515, USA

**Keywords:** flavonoids, apigenin, chrysin, metabolomics, therapeutic prediction

## Abstract

The prominent flavonoids apigenin and chrysin have been demonstrated to have systemic benefits. Our previous work was first to establish the impact of apigenin and chrysin on cellular transcriptome. In the current study, we have revealed the ability of apigenin and chrysin to alter the cellular metabolome based on our untargeted metabolomics. Based on our metabolomics data, both these structurally related flavonoids demonstrate diverging and converging properties. Apigenin demonstrated the potential to possess anti-inflammatory and vasorelaxant properties through the upregulation of intermediate metabolites of alpha-linolenic acid and linoleic acid pathways. Chrysin, on the other hand, exhibited abilities to inhibit protein and pyrimidine synthesis along with downregulation of gluconeogenesis pathways based on the altered metabolites detected. Chrysin-mediated metabolite changes are mostly due to its ability to modulate L-alanine metabolism and the urea cycle. On the other hand, both the flavonoids also demonstrated converging properties. Apigenin and chrysin were able to downregulate metabolites involved in cholesterol biosynthesis and uric acid synthesis, namely 7-dehydrocholesterol and xanthosine, respectively. This work will provide understanding regarding the diverse therapeutic potential of these naturally occurring flavonoids and help us in curbing an array of metabolic complications.

## 1. Introduction

Foods are a direct link to our environment. The global population relies mostly on naturally available foods from plant and animal sources [1,2,3]. Polyphenols are naturally occurring bioactive compounds presents in vegetables, fruits, and other plant parts that humans have been consuming for years [4,5]. In recent decades, chemists have developed strategies and tools to isolate the pure individual bioactive component from a plant part or the whole plant. This is important for understanding the physiological impact of that individual component. Apigenin and chrysin are two structurally related polyphenols belonging to the flavonoid family that are present in fruits, leaves, and vegetables [6,7]. In the available literature, the medical benefits of apigenin and chrysin are well described [4,5,6,8]. The rationale for using these agents in the available reports published to date has been more of a “pick and choose” strategy. Most of the published work has only portrayed the beneficial effects associated with apigenin and/or chrysin in the area of cardiovascular disease, cancer, or neurodegeneration [4,5,6,8]. From a pharmacological perspective, it is very crucial to remember that these natural agents are not specific ligands and could regulate an array of targets, resulting in a landscape shift in cellular signaling and function. When we consume these agents through foods, we cannot tightly control the dose. There are dose-specific and dose-dependent pharmacological and toxicological effects that are entirely unknown and need to be identified.

In our previous work, using a transcriptomic approach, we identified that both apigenin and chrysin were able to downregulate transcripts involved in the cholesterol biosynthesis pathway while promoting the ketogenic pathway [7]. In our current work using an untargeted metabolomics approach, we have corroborated our past findings, and have demonstrated that a penultimate metabolite in the cholesterol biosynthesis pathway is downregulated. Simultaneously, the compounds also exhibited some diverse features as demonstrated by the altered metabolite levels. Apigenin was able to upregulate alpha-linolenic acid and linoleic acid pathways, whereas chrysin was able to downregulate nucleic acid, and protein biosynthesis and gluconeogenesis pathways. Enhanced alpha-linolenic and linoleic acid pathways could lead improvement of cardiovascular and cerebrovascular function [9,10,11,12,13]. Apigenin could potentially serve as an ideal agent to treat cardiovascular and cerebrovascular potential due to this reason. Dysregulated transcription and translational processes contribute towards tumor progression, and inhibitors of both transcription and translation have been a major highlight in developing cancer therapeutics [14,15]. Gluconeogenesis is a major contributing factor for hyperglycemia in type 2 diabetes, and downregulators of gluconeogenesis pathways are being studied [16,17,18,19,20,21]. In our study, chrysin has demonstrated the dual potential of downregulating pathways of gluconeogenesis with transcription and translation, making it a potential therapeutic agent for treating complications associated with type 2 diabetes and cancer.

Based on our current omics-based approach, we were able to predict the potential application for these two beneficial flavonoids. Our study will not only reveal the potential benefits but also the associated risks, which could help us in the judicious use of these bioactive agents. This work will reveal a plethora of information regarding these two polyphenols and will help us in determining the therapeutic relevance of these agents along with revealing their suitability for specific indications.

## 2. Results

### 2.1. Apigenin and Chrysin Treatment Altered the Whole Cell Metabolome in MEF Cells

**Figure 1:**
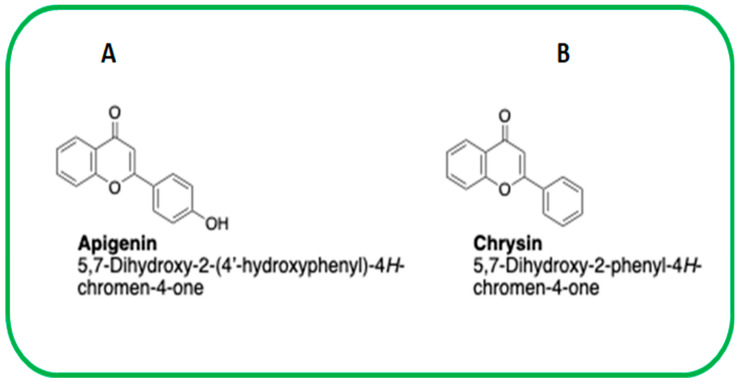
IUPAC nomenclature for structurally related flavonoids. (**A**) Apigenin is a 5,7-Dihydroxy-2-(4′-hydroxyphenyl)-4*H*-chromen-4-one and (**B**) chrysin is a 5,7-Dihydroxy-2-phenyl-4*H*-chromen-4-one.

The two closely related flavonoids, apigenin and chrysin (Figure 1A), altered the cellular metabolome in MEFs. Metabolites obtained from both negative and positive ion mode (ESI− and ESI+) were analyzed using principal components analysis (PCA), partial least squares discriminant analysis (PLS-DA) or orthogonal partial least squares discriminant analysis (OPLS-DA) to reveal the different metabolites formed following apigenin and chrysin treatments in comparison with the control treatment. A clear distinction was evident between the cluster of formed metabolites in the apigenin- and chrysin-treated groups in comparison with the control groups in both ESI− (Supplementary Figure S1) and ESI+ mode (Supplementary Figure S3). A volcanic plot of metabolite data sets validated the findings that apigenin and chrysin can make that clear-cut distinction in the metabolite changes. Univariate analysis of volcanic plot of metabolite sets when expressed as a log2 fold change revealed that apigenin was able to upregulate and chrysin was able to downregulate some of the major metabolite markers within the fibroblast cells, as exhibited by data sets from both negative (Supplementary Figure S2) and positive (Supplementary Figure S4) ion modes. The increase or decrease in metabolite levels, as depicted by data points in the volcanic plot, (colored) pink, is in the significant range. These data sets reveal the ability of these closely related flavonoids in regulating cellular metabolome.

### 2.2. Alpha-Linolenic Acid and Linoleic Acid Metabolism Emerged as the Major Metabolic Pathways Specifically Regulated by Apigenin

**Figure 2:**
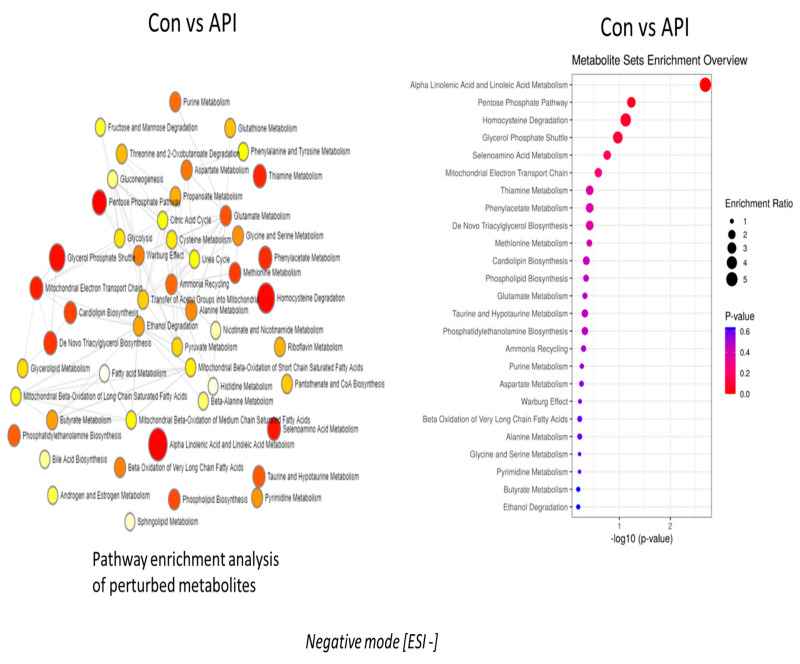
Pathway enrichment analysis of altered metabolites in mouse embryonic fibroblasts following apigenin treatment. Following 24 h of apigenin treatment, there were numerous metabolite pathways, which were altered in negative ion mode.

**Figure 3:**
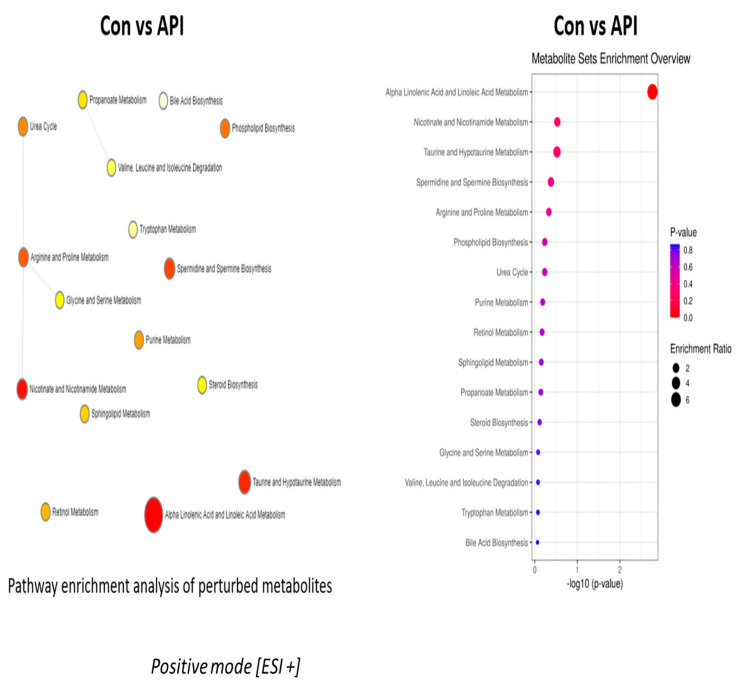
The figure depicts pathway enrichment analysis and the metabolic pathways that were significantly altered in positive ion mode following apigenin treatment.

**Figure 4:**
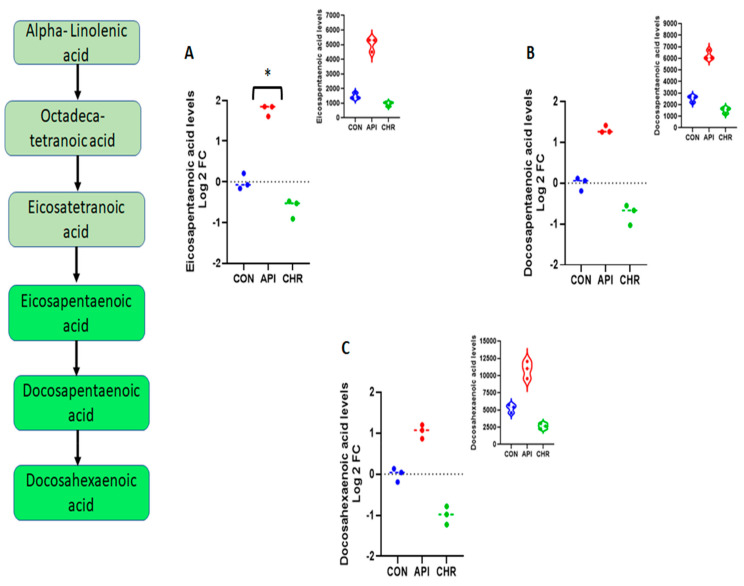
Analysis of components of the alpha-linolenic acid pathway regulated by apigenin. The metabolites belonging to the alpha-linolenic acid pathway that appeared in the analysis panel were: (**A**) eicosapentaenoic acid, (**B**) docosapentaenoic acid, and (**C**) docosahexaenoic acid. The raw values plotted on the upper inserts and the raw values converted to a log2 fold change and plotted using GraphPad Prism were used in the main figure. Significance was determined based on * *p* < 0.05 and with a log2 fold change difference of +/− 1.5 fold change.

**Figure 5:**
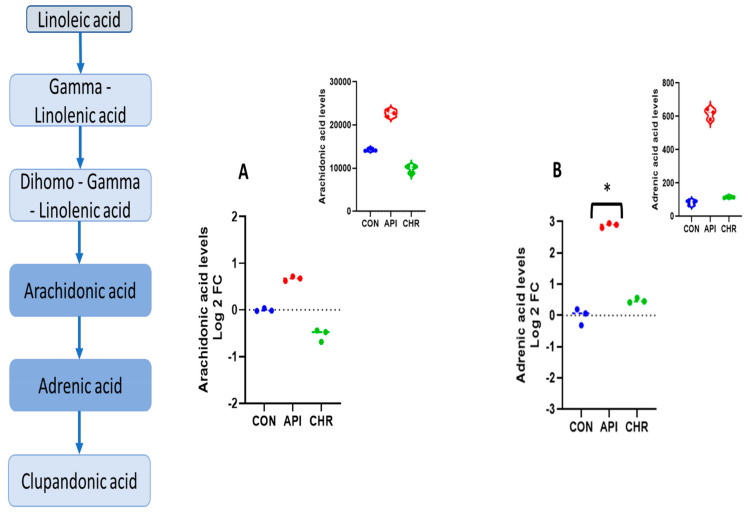
Metabolites of the linoleic acid pathway regulated by apigenin. In the linoleic acid pathway, the metabolites that were differentially regulated by apigenin were: (**A**) arachidonic acid, and (**B**) adrenic acid. The upper inserts represent the raw values. The log2 fold change values were plotted using GraphPad and significance set at * *p* < 0.05 with a +/− 1.5 fold change.

Pathway enrichment analysis of altered metabolites revealed that the alpha-linolenic and linoleic acid pathways were the prominently regulated pathways by apigenin unanimously in both negative and positive ion mode, as depicted in Figure 2 and Figure 3, respectively. The enrichment ratio, along with the *p*-value reached at significant levels for the metabolites, were selected. Specific analysis of metabolites belonging to these two major pathways regulated by apigenin revealed that eicosapentaenoic acid, docosapentaenoic acid, and docosahexaenoic acid were the major ones. When comparing metabolites, the parameters were set to a limit of >±1.5 fold change (increase/decrease) as per the log2 fold change values, with a statistical significance set at *p* < 0.05. Eicosapentaenoic acid levels in apigenin-treated MEFs showed a significant increase, with log2 fold change values >1.7 fold compared with control groups and even chrysin-treated groups based on the values from the negative ion mode (Figure 4A). Docosapentaenoic acid and docosahexaenoic acid exhibited only >1.3 and >1.04, respectively, as per the log2 fold change expressed in negative ion mode in comparison with both control and chrysin treatment groups (Figure 4B,C) and did not fulfill the selection criteria. For metabolites in the linoleic acid pathway, the metabolites that appeared in the list were arachidonic acid and adrenic acid. The changes in arachidonic levels were statistically significant (*p* < 0.05) but the log2 fold change did not reach 1.5 fold, rather it was a 0.671 fold increase (Figure 5A). Interestingly, adrenic acid reached a > 2.88 log2 fold change (increase) along with the statistical significance (*p* < 0.05) compared with both the control and chrysin-treated groups (Figure 5B). The original peak values for the metabolites are available as upper inserts in these figures.

### 2.3. Alanine Metabolism and Urea Cycle Are the Major Metabolic Pathways Specifically Controlled by Chrysin

**Figure 6:**
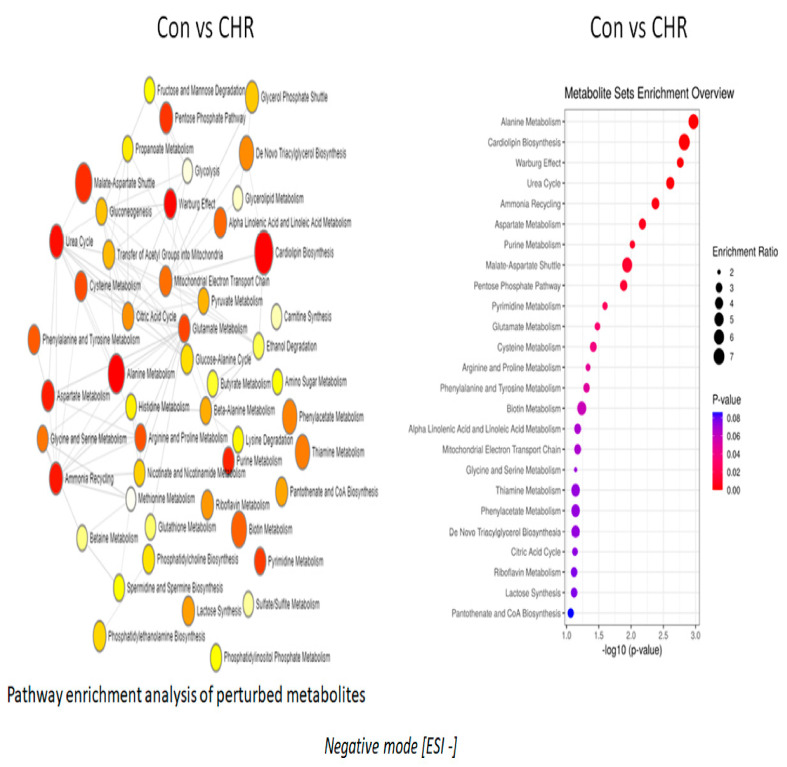
Metabolic pathways regulated by chrysin treatment in negative ion mode. The pathway enrichment analysis and enrichment ratio in the negative ion mode are illustrated in this figure.

**Figure 7:**
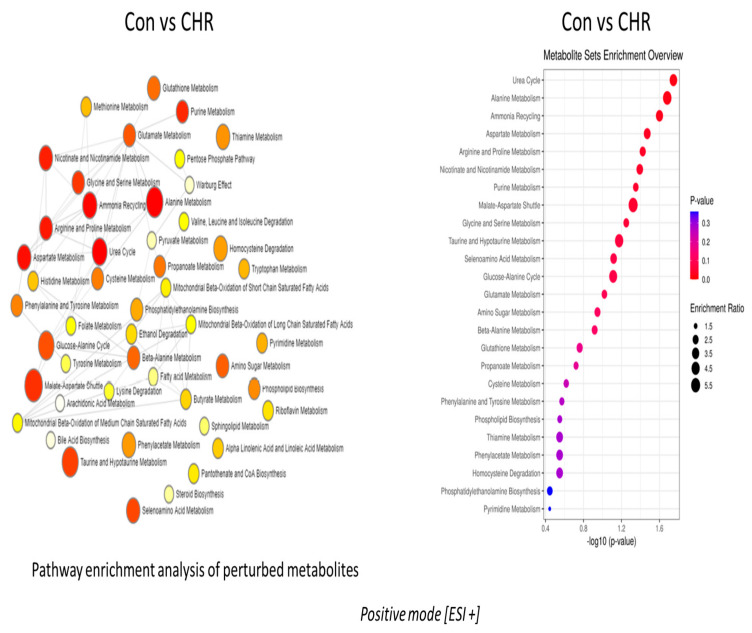
Chrysin-regulated pathways in positive ion mode. The enrichment pathway analysis along with the enrichment ratio in the positive ion mode are clearly depicted.

**Figure 8:**
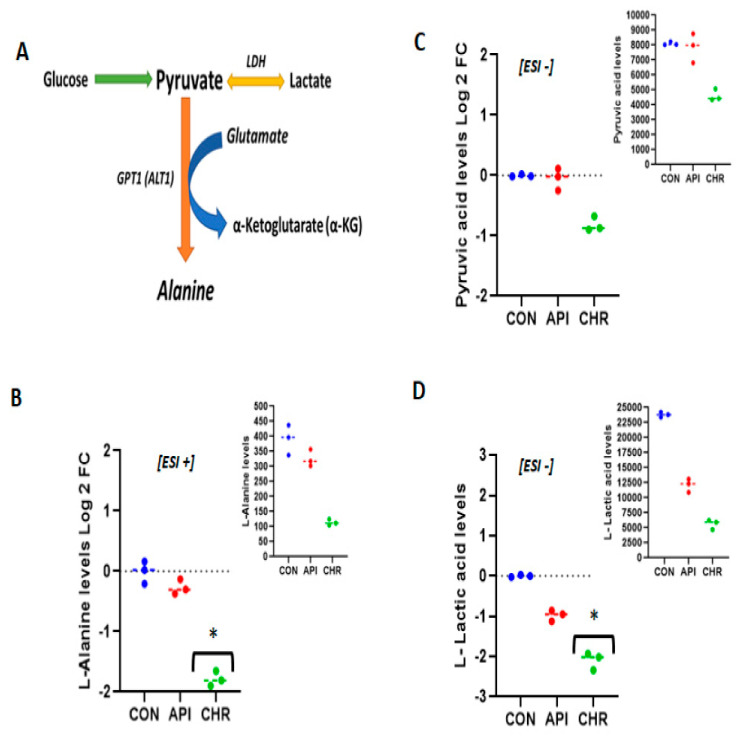
Regulation of alanine metabolism by chrysin: The schematics for alanine regulation in non-hepatic cells are represented. (**A**) The source of alanine is from either of the glucose metabolites—pyruvate or lactate. The formed alanine is then effluxed of the cell. (**B**) Represents L-alanine raw values (upper insert) and log2 fold change values. The log2 fold change values for pyruvic acid and lactic acid, which are the acidic forms for pyruvate and lactate with their respective raw values (inserts), are represented in Figure 3 panel (**C**,**D**), respectively * *p <* 0.05.

**Figure 9:**
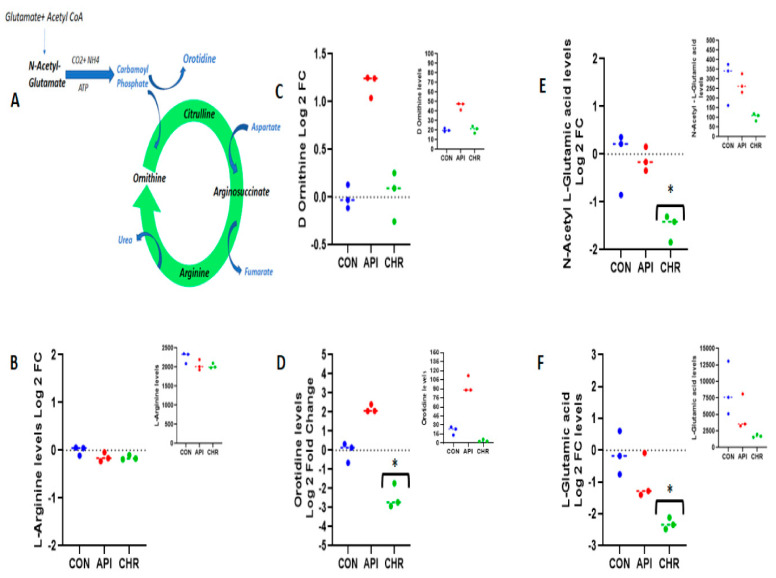
Chrysin-mediated regulation of metabolites in the urea cycle: Based on the metabolite panel, the urea cycle was the other top regulated metabolic pathway. (**A**) Glutamate and acetyl CoA act as major substrates for the formation of N-acetyl glutamate and finally carbamoyl phosphate, which is the precursor for substrates in the urea cycle including citrulline, arginine, and ornithine. The raw values (upper inserts) and the log2 fold change values of (**B**) L-arginine, (**C**) D-ornithine, and (**D**) orotidine are expressed, respectively. The protonated forms of N-acetylated glutamate and glutamate, N-acetyl L-glutamic acid, and L-glutamic acid with the raw values (upper inserts) and calculated log2 fold change values are represented in (**E**,**F**), respectively * *p <* 0.05.

The metabolite sets that emerged as the top regulated ones by chrysin in negative (Figure 6) and positive (Figure 7), as revealed by pathway enrichment analysis, were the urea cycle and alanine metabolism, respectively. L-Alanine metabolism is tissue specifically regulated. In non-hepatic cells, such as in MEFs, L-alanine can be formed from pyruvate via glucose or lactate sources (Figure 8A). The formed L-alanine either can be released into the circulation, which can be taken up by the liver for the process of gluconeogenesis, or can be used for the synthesis of proteins. It can also serve as an amino acid source for ATP generation in normal tissues and also in cancerous tissues. When measured, L-alanine levels decreased >1.7 fold (−1.78) as expressed by the log2 fold change with the statistical significance in comparison with the control and apigenin-treated MEFs (Figure 8B). We also analyzed the levels of pyruvate and lactate. Pyruvate expressed in the protonated form as pyruvic acid did not reach the cut-off criteria (< or >1.5 fold), but rather only demonstrated a 0.815-fold decrease (Figure 8C). Unlike pyruvic acid, lactic acid levels demonstrated >2.09 fold decrease (−2.09) as expressed by log2 fold change levels in comparison with the control and apigenin-treated groups (Figure 8D). The levels of L-alanine, pyruvic acid, and lactic acid correlated well, demonstrating the channelization of this metabolic pathway. Regarding the urea cycle, the intermediates within the urea cycle, L-arginine and D-ornithine, did not exhibit major change compared with the control or apigenin-treated groups (Figure 9B,C). A major precursor or substrate provider for the urea cycle is carbamoyl phosphate, which has two major fates—either helping in the formation of citrulline or taking an alternative path, forming orotidine, as shown in Figure 9A. Carbamoyl phosphate is formed from N-acetyl glutamate in the presence of carbon dioxide, ammonium ion, and ATP. N-acetyl glutamate is formed by the combination of glutamate and acetyl coA (Figure 9A). In the absence of any change in the metabolites/substrates of urea cycle, we measured the levels of orotidine, N-acetyl glutamic acid, and glutamic acid; the protonated forms of N-acetyl glutamate and glutamate were the ones that appeared in the metabolite list. Interestingly, there was over a 2.457 fold level decrease in orotidine (>−2.457 fold, Figure 9D), which correlated well with over a 1.52 decrease (>−1.52 fold, Figure 9E) for N-acetyl glutamine and over a 2.30 fold decrease (>−2.30 fold, Figure 9F) for L-glutamic acid, which are the upstream metabolites of orotidine as expressed by log2 fold change values. Orotidine is a major precursor for pyrimidine synthesis and is known to generate pyrimidine bases. In our metabolite list the downstream metabolite of orotidine and a pyrimidine base, uracil, demonstrated a significant change, with a log2 fold change over −2.675 (Supplementary Figure S7). The nucleotide form of uracil and cytosine, uridine monophosphate and cytidine monophosphate, also demonstrated significant changes with log2 fold change values of >−2.17 and >−1.89, respectively (decrease) (Supplementary Figure S8).

The peak/raw values for the metabolites belonging to different groups are represented as upper inserts in each figure.

### 2.4. Apigenin and Chrysin Demonstrated Similarity in Downregulating Metabolites Involved in Cholesterol and Uric Acid Biosynthesis Pathways

**Figure 10:**
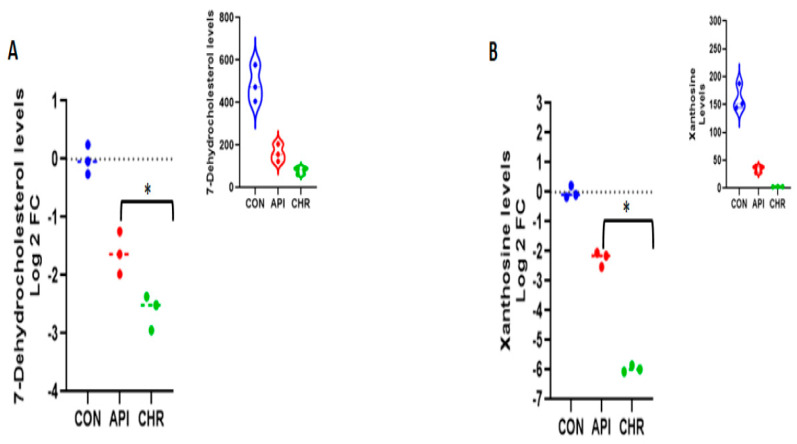
Downregulation of cholesterol and uric acid pathways by apigenin and chrysin: Both apigenin and chrysin demonstrated to have a suppressive effect on the cholesterol biosynthetic pathway and uric acid pathway based on the major metabolites belonging to these pathways. (**A**) 7-Dehydrocholesterol and (**B**) xanthosine levels as determined by raw values (upper inserts) and log2 fold change values as expressed in respective figures with a significance of * *p* < 0.05.

Along with the divergent properties demonstrated by both flavonoids, apigenin and chrysin, in regulating specific metabolites, they both also exhibited some commonality. Major metabolites that were regulated in a similar manner by both apigenin and chrysin were 7-dehydrocholesterol and xanthosine. Both 7-dehydrocholesterol and xanthosine are the major intermediate substrates/metabolites in cholesterol and uric acid biosynthesis pathways. 7-Dehydrocholesterol demonstrated over a 1.618 fold (>−1.618) and 2.605 fold (>−2.605) decrease as expressed by the log2 fold change values for apigenin and chrysin, respectively (Figure 10A). Xanthosine demonstrated a decrease of over 2.23 fold (>−2.23) and over 5.963 fold (>−5.963) for apigenin and chrysin, respectively, as expressed by the log2 fold change values (Figure 10B). The original peak values for the metabolites are expressed as upper inserts inside the figures.

## 3. Discussion

There is not much known about the impact of apigenin and chrysin on the cellular metabolome. Rather than directly advocating for the natural compounds of interest in treating a specific disease, it is important to understand in depth the overall impact of using these flavonoids at the cellular level. Our previous work demonstrated the influence of apigenin and chrysin at the cellular transcriptome level [7]. Current work has furthered the information by revealing the impact these compounds have on the cellular metabolome. Along with their ability to turn on or off certain transcripts, as demonstrated by our previous work, this study with the application of untargeted metabolomics revealed their ability to change the metabolite levels inside the cell. The data have revealed an in-depth knowledge regarding how these compounds could influence cellular function by altering various metabolic pathways. In addition, it is crucial to consider the pros and cons for these agents before we advocate them for specific conditions. Apigenin and chrysin are structurally similar and related flavonoids but differ in chemical structure by having one 4-hydroxyl group in the 2-phenyl for apigenin compared with chrysin as, described in Figure 1.

Even with a difference of just one hydroxyl group (Figure 1), there is a major divergence in the metabolic properties possessed and the metabolites regulated by these two compounds (Supplementary Figure S5). Interestingly, in both negative and positive ion mode metabolite detection, the alpha-linolenic acid and linoleic acid metabolic pathways are the crucial ones regulated by apigenin in a significant manner, with a high enrichment ratio (Figure 2 and Figure 3). This was a notable observation not only against the control group but also against the chrysin-treated groups (Supplementary Figure S6). Based on these observations we could deduce that apigenin could be an ideal agent for activating the alpha-linolenic acid and linoleic acid pathways. The protective role of alpha-linolenic acid and linoleic acid in cardiovascular diseases and neurodegenerative diseases is well established but we have rarely perceived agents that can channelize the intracellular lipid metabolism [22,23,24,25,26,27,28,29,30]. Among the metabolites regulated in these pathways, EPA, DPA, and DHA demonstrated changes, but only EPA levels were significant with a >1.5 fold change as expressed by log2 FC values. EPA levels increased >1.7 fold, whereas DPA and DHA levels only increased > 1.3 and >1.04 fold, respectively. It is a clinically proven fact based on data from numerous studies that a prolonged DHA increase could enhance the LDL-cholesterol (which is considered the bad cholesterol) levels in subjects [31,32]. In contrast, EPA provision is known to decrease the total cholesterol and triglyceride concentrations and improve cardiovascular health. In recent years, the only EPA-containing FDA-approved drug (Vascepa) has demonstrated incredible cardiovascular benefits, which reaffirms this claim [33,34]. The uniqueness about our findings is that apigenin can trigger the biochemical pathways involving the alpha-linolenic acid pathway, leading to more EPA than any other metabolites, which could be protective not only for the cardiovascular system but also for systemic health. The other metabolic pathway top regulated by apigenin is the linoleic acid pathway, as per our findings. In this pathway, both arachidonic acid and adrenic acid were upregulated, but adrenic acid was the only metabolite that demonstrated a log2 FC of > 1.5 (> 2.88) in comparison with arachidonic acid, which also changed > 0.671 (log2 FC). Adrenic acid is also known as 7,10,13,16-docosatetraenoic acid, which is an omega (ώ)-6 polyunsaturated fatty acid [35]. The protective roles for adrenic acid include endothelial-derived relaxation factor, as demonstrated in the bovine coronary artery model and adrenal cortical arteries, and as an anti-inflammatory agent inhibiting leukotriene synthesis (LTB4) in neutrophils using the murine model of peritonitis and arthritis [35,36,37]. Apigenin, by enhancing endogenous adrenic levels without significantly elevating arachidonic acid, could potentially enhance vasorelaxant and anti-inflammatory effects in cardiovascular and other systems but it needs further testing in vivo. These findings for the first time provide a clear insight into the multiple protective signaling networks turned on by the apigenin-mediated major metabolites EPA and adrenic acid.

With regard to chrysin, L-alanine metabolism was the most regulated pathway in negative ion mode and the urea cycle in positive ion mode. Hepatic tissue assimilates alanine secreted by other tissues into the circulation and converts it into glucose through the process of gluconeogenesis and make use of excess alanine as the raw material for the synthesis of proteins [38,39,40]. When there is an upregulation of L-alanine metabolism, it could potentially lead to enhanced gluconeogenesis, as seen during insulin resistance, cancer [39,41], and diabetes [41,42,43]. In addition, excess protein biosynthesis is another hallmark of metabolic syndrome and cancer [42,43]. By curbing L-alanine provision to the liver, we could limit the upregulation of gluconeogenesis and the trigger to synthesis excess proteins by the liver, therefore reducing hepatic stress. These phenomena (excess gluconeogenesis and excess protein synthesis) are very prevalent in insulin resistance, diabetes, and cancer. By curbing these pathways, we could potentially limit metabolic complications associated with diabetes and cancer. Besides L-alanine, the other top major metabolic pathway regulated by chrysin in positive ion mode was the urea cycle. Interestingly, as mentioned in our results section, no significant changes were noted in the levels of intermediate metabolites in the urea cycle, which came up in our analyte list, arginine and ornithine. An alternative fate for carbamoyl phosphate, the precursor for intermediates in the urea cycle, is to form orotidine, which is a known precursor for pyrimidine nucleotides [44,45]. Most malignancies have been associated with excess pyrimidine nucleotide synthesis, and inhibiting this biochemical process has been considered a major strategy to combat malignancies [46,47,48]. Both orotidine and its predecessor, N-acetyl glutamate, have been demonstrated to be downregulated by chrysin treatment not only in comparison with the control group but even with the apigenin-treated groups. We also evaluated the levels of downstream metabolites of orotidine, the pyrimidine base, uracil (Supplementary Figure S7), and its nucleotide form, uridine monophosphate, along with another pyrimidine nucleotide analog, cytidine monophosphate (Supplementary Figure S8). These downstream effects confirm chrysin’s ability to regulate pyrimidine biosynthesis. The unique ability of chrysin to downregulate orotidine, a pyrimidine precursor, demarcates it from its flavonoid counterpart in the treatment of cancer and associated complications. By influencing both L-alanine- and orotidine-mediated pyrimidine nucleotide synthesis pathways, chrysin emerges as an ideal candidate that could curb gluconeogenesis, and prevent excess protein and pyrimidine synthesis, as seen with cancer [42,47,48].

**Figure 11:**
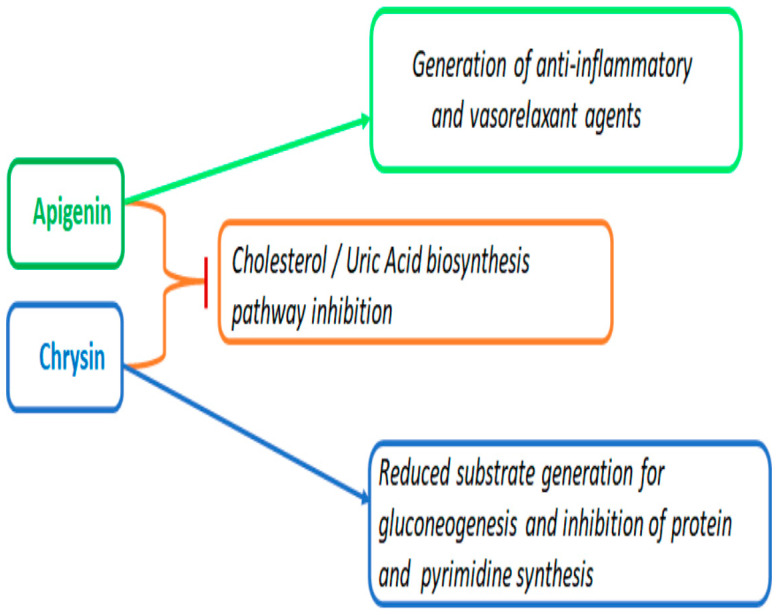
Summary diagram demonstrating the distinct and similar metabolic pathways regulated by apigenin and chrysin. Apigenin, through the regulation of the alpha-linolenic and linoleic acid pathways, generates endogenous anti-inflammatory and vasorelaxant metabolites. On the other hand, chrysin, by regulating alanine and urea cycles, suppresses the gluconeogenesis, protein synthesis, and pyrimidine synthesis pathways. Both apigenin and chrysin demonstrate converging effects by inhibiting the generation of metabolites involved in biosynthesis of cholesterol and uric acid pathways.

Even with the existing divergence, we have observed some converging features in these structurally related flavonoids. Our previous work, based on transcriptomic analysis, was the first to show that both apigenin and chrysin possess hypocholesterolemic properties by downregulating multiple enzymes in the mevalonate pathway [7]. In the current work, we observed consolidating evidence that both apigenin and chrysin were able to downregulate 7-dehydrocholesterol, the penultimate metabolite in the cholesterol biosynthesis pathway. Based on the observed results, chrysin was able to downregulate in a robust manner, even compared with apigenin **(−2.6 vs. −1.6)**. Interestingly, another novel target that was downregulated by both apigenin and chrysin was xanthosine **(−2.23 and −5.9)** in comparison with the control. Both cholesterol and uric-acid accumulation has been reported to be an initiator of metabolic complications in cardiovascular pathologies and cancer [49,50,51]. Uric acid is also the biochemical end product of purine metabolism, which is elevated during both cancer and cardiovascular complications [52,53]. Agents to lower hypocholesterolemia have been considered a major therapeutic strategy for treating cardiovascular complications and have been studied for over a decade [54,55]. Recently, NIH-funded clinical trials—the Colchicine Cardiovascular Outcomes Trial (COLCOT) and Low-Dose Colchicine (LoDoCo) —have brought to light the significance of inhibiting uric acid using colchicine following cardiac ischemic conditions [56,57]. Apigenin and chrysin, with their ability to simultaneously inhibit cholesterol and uric acid, could serve as ideal agents to curb metabolic complications found during cancer and cardiovascular diseases (Figure 11).

## 4. Materials and Methods

### 4.1. Cell Culture and Treatments

Mouse embryonic fibroblasts (MEFs) purchased from Lonza, Inc. (Walkersville MD, USA) with Cat#M-FB-481 were employed for our experiments. Healthy passages from 2 to 5 were used for the different treatment groups. The cells were cultured and passaged in Dulbecco’s modification of Eagle’s Medium (DMEM) (Corning®, USA, Manassas, VA). DMEM comprising high glucose (4.5 g/L) was filtered along with 10% fetal bovine serum (FBS00), 500 mL, USDA-Origin, Neuromics, MN, USA, and 1.5% penicillin/streptomycin and amphotericin from VWR, Avantor, USA. During treatment, the serum-containing medium was replaced with a serum-free 1g/L glucose-containing (DMEM) medium supplied with only 1.5% penicillin/streptomycin and amphotericin. The cells were subjected to only serum-free 1g/L glucose containing (DMEM) medium (controls) or incubated with 25 μM apigenin in 1 g/l glucose containing DMEM or 25 μM chrysin in 1 g/L glucose containing DMEM for 24 h before they were pelleted, and shipped at freezing temperatures using dry ice to perform untargeted metabolomics. Each group had n = 3 replicates.

### 4.2. Sample Preparation

The untargeted metabolomics service was performed at our outsourcing facility (Creative Proteomics, NY, USA). In brief, the shipped cell pellets were thawed, and 80–85% methanol was added to cover the pellets (750–1000 µL). These sample sets were subjected to ultrasound-based extraction at a steady temperature set at 4 °C for approximately 30 min. Following ultrasound exposure, samples were kept at −40 °C for at least an hour. After the cold exposure, samples were removed, vortexed well for 30 s, and then centrifuged at a speed of over 12000 rpm at 4 °C for at least 12–15 min. A clear supernatant from the top layer of approximately 200µL and DL-O-Chloro-phenylalanine at a concentration of 140 μg/mL made into 2–5 µL was transferred to a vial for LC-MS analysis.

### 4.3. Chemical Structures

The chemical structures with the IUPAC names were generated using the software ChemDraw Prime v19.1 from Perkin Elmer.

### 4.4. UPLC-TOF-MS Technology

Ultra performance liquid chromatography (UPLC) with time-of-flight mass spectrometry (ESI-TOF-MS) was performed at our outsourcing facility by a well-established method [58,59]. In brief, the sample separation was performed using UltiMate 3000LC combined with Q Exactive mass spectrometry (Thermo) followed by screening with ESI-MS. The LC system is a combination of a two-system unit with Thermo hyper gold C18 (100×2.1mm 1.9 μm) combined with the UltiMate 3000LC system. The mobile phase comprises two solvents—solvent A and solvent B. Solvent A comprises 0.1% formic acid, 5% acetonitrile, and water, and solvent B is a mixture of 0.1% formic acid and acetonitrile with a gradient elution of 0–1.5 min, 0–20% B; 1.5–9.5 min, 20–100% B; 9.5–14.5 min, 100% B; 14.5–14.6 min, 100–0% B; 14.6–18.0 min, 0% B. The flow rate for the mobile phase was fixed at 0.3 mL/min. The column temperature was maintained at 40°C, and the sample manager temperature was set at 4 °C. Mass spectrometry parameters in ESI+ and ESI- modes were set. For positive ion mode (ESI+), the experimental parameters were the following: heater temperature set at 300 °C, flow rate of the sheath gas set at 45 arb, auxiliary gas flow rate set at 15 arb, sweep gas flow rate set at 1 arb with a spray voltage of 3.0 kV, capillary temperature set at 350 °C, and the S-Lens RF level adjusted to 30%.

### 4.5. Statistical Analysis

The analysis was performed using a well-established previously published statistical method [59,60]. Following the acquisition of the raw data, these data are aligned with the aid of Compound Discover using the 3.0 system from Thermo based on their m/z ratio and the retention times of ion signals. The emerging ions from both the positive (ESI+) and negative (ESI-) ion modes are fused before importing into the SIMCA-P program (version 14.1) for multivariate analysis. A preliminary unsupervised method is employed for principal components analysis (PCA) for visualization of data and for the identification of outliers. The data sets are then subjected to a supervised version of regression modeling using partial least squares discriminant analysis (PLS-DA) or orthogonal partial least squares discriminant analysis (OPLS-DA) to identify the target metabolites. The filtered-out metabolites are confirmed by combining the obtained results with those of variable importance in projection (VIP) values. The VIP values > 1.5 and *p* values < 0.05 based on the *t*-test are taken into consideration. The quality of data fit is then explained with the help of R2 and Q2 values. R2 indicates the variance and denotes the quality of the fit explained in the model. Q2 indicates the variance in the data with the model’s predictability.

From the obtained raw values from three replicates for each group, average values were calculated, and then fold change and log2 fold change were calculated. A log2 fold change of > +/−1.5 with a statistical significance as indicated by *p* < 0.05 was considered as a significant change. The calculated values were analyzed using GraphPad Prism software to evaluate the statistical significance of the test and difference between groups and plotted.

## 5. Conclusions

To conclude, our work based on an untargeted metabolomics approach reveals the unique properties of two closely related flavonoids. Currently we do not have much information on how these agents act at the cellular level, dissecting their effects on the cellular metabolome. This work will lay the foundation for future studies involving apigenin and chrysin in understanding the pharmacological properties and specific influence of these agents on the cellular metabolic landscape. As far as the **limitations** of this study are concerned, our predictions are based on untargeted metabolomics from an in vitro model system, and the predictions on the systemic effects are based on extrapolations. We recommend that this work should be followed up with studies involving in vivo model systems to identify the correct dose and toxicity when treating metabolic complications associated with cardiovascular disease or cancer. Both apigenin and chrysin have demonstrated high potential to emerge as therapeutic agents that can help in curbing metabolic diseases with feasibility, and with predictable and minimal adverse effects.

## Data Availability

All the raw/original data will be available for public in Metabolomics Workbench.

## Supplementary Materials

The following supporting information can be downloaded at: https://www.mdpi.com/article/10.3390/ijms24044066/s1.
