# Supplementary material for: Untargeted Metabolomics Based Prediction of Therapeutic Potential for Apigenin and Chrysin
Source: Int J Mol Sci. 2023 Feb 17;24(4):4066. doi: 10.3390/ijms24044066 (PMC9967419; doi:10.3390/ijms24044066)
Supplement: Supplementary file 1 [file ijms-24-04066-s001.zip › ijms-2154964-supplementary.pdf]

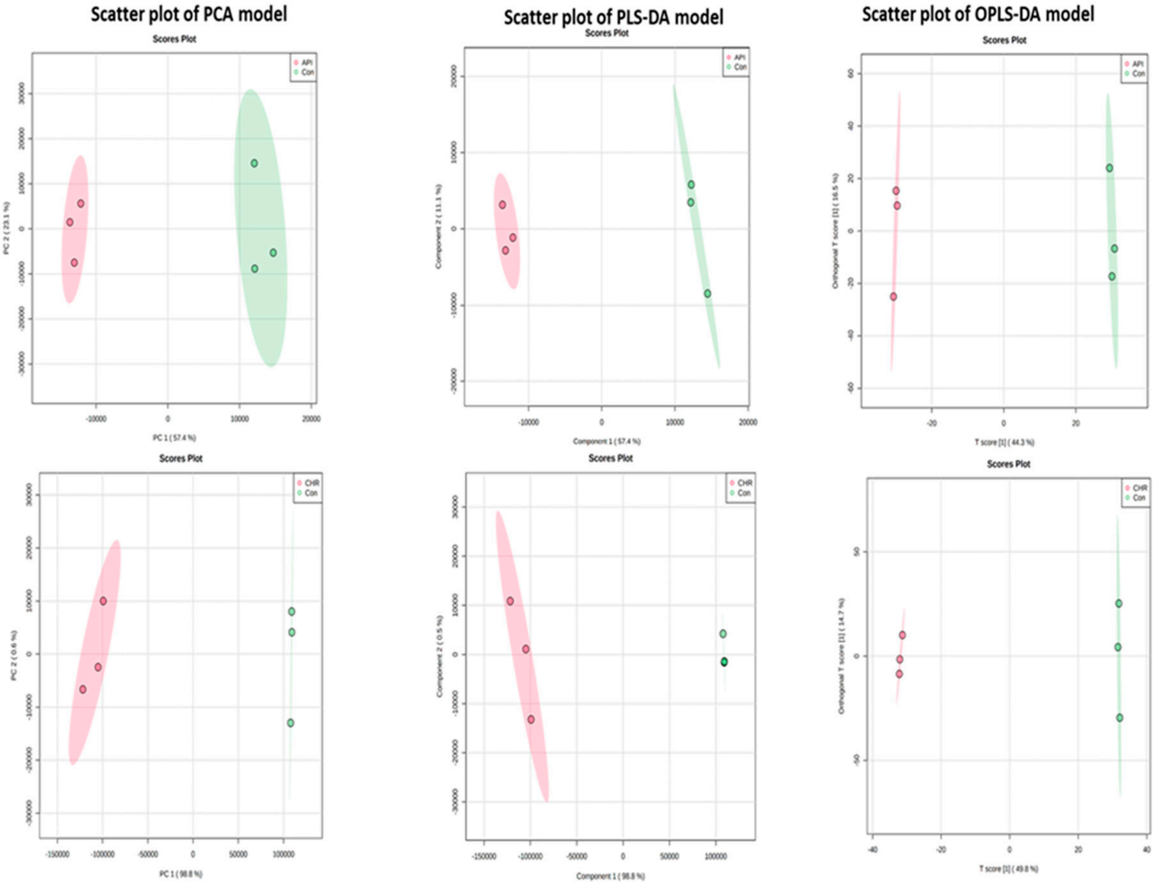

Scheme S1. Metabolic profiling of mouse embryonic fibroblasts following apigenin and chrysin treatment for 24 hours. Scatter plots following Principal Components Analysis (PCA), Partial Least Squares Discriminant Analysis (PLS-DA) and Orthogonal Partial Least Squares Discriminant Analysis (OPLS-DA) for both Apigenin and Chrysin in negative ion mode is depicted in the figure.

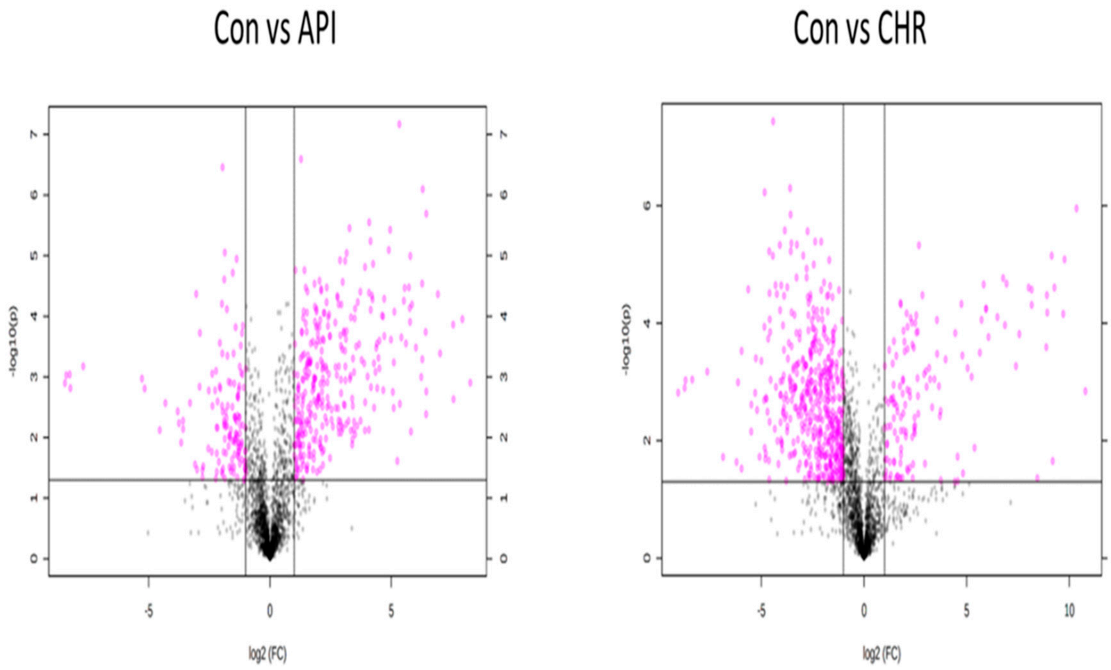

Scheme S2. The volcanic plot based metabolic profiling for negative ion mode for both apigenin and chrysin.

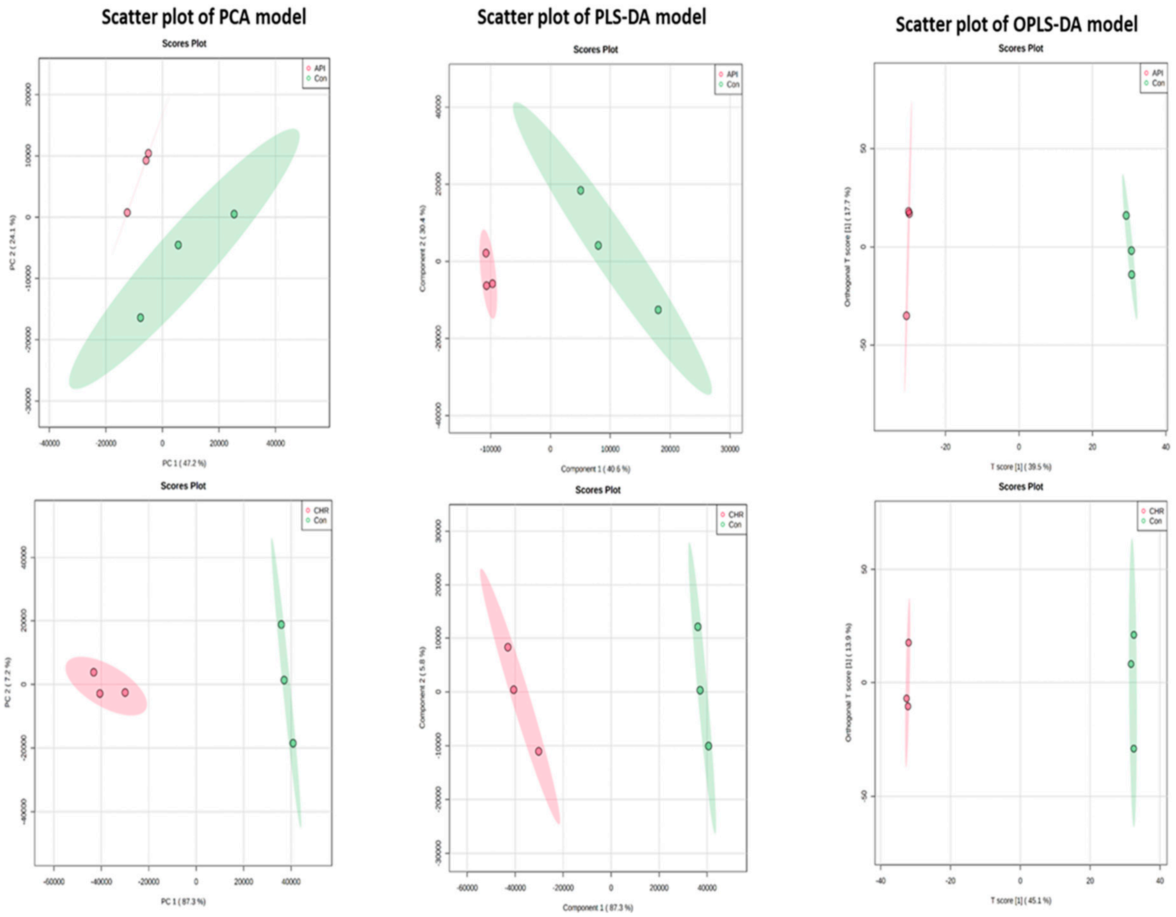

Scheme S3. The metabolic profiling scatter plots for Principal Components Analysis (PCA), Partial Least Squares Discriminant Analysis (PLS-DA) and Orthogonal Partial Least Squares Discriminant Analysis (OPLS-DA) in positive ion mode for both Apigenin and Chrysin.

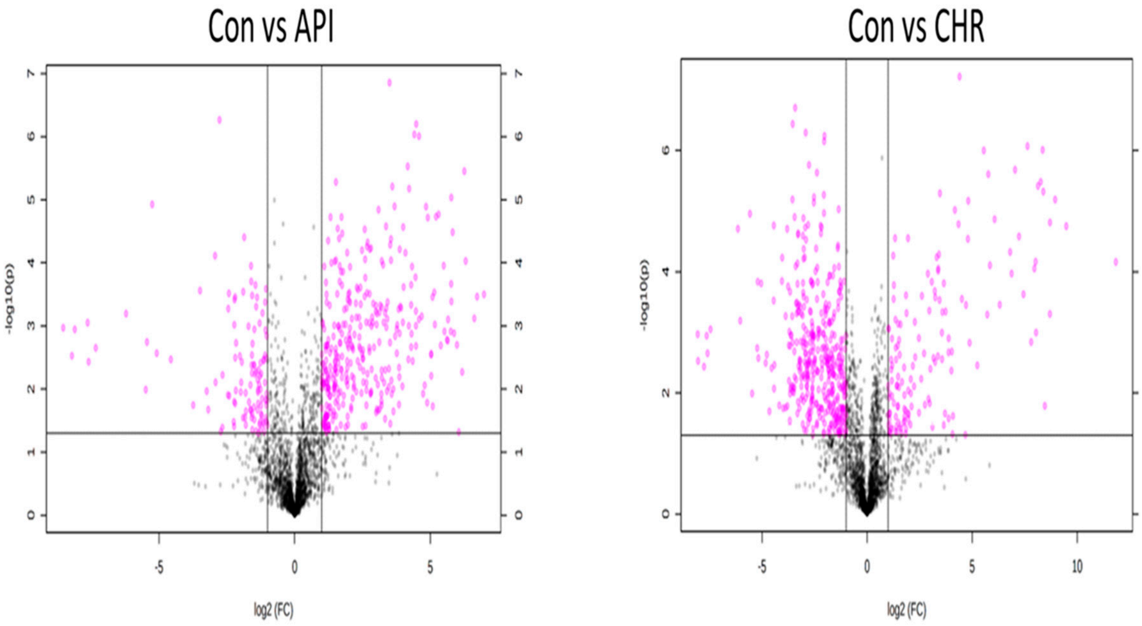

Scheme S4. Metabolic profiling using the volcanic plot in negative ion mode for apigenin and chrysin is illustrated in this figure.

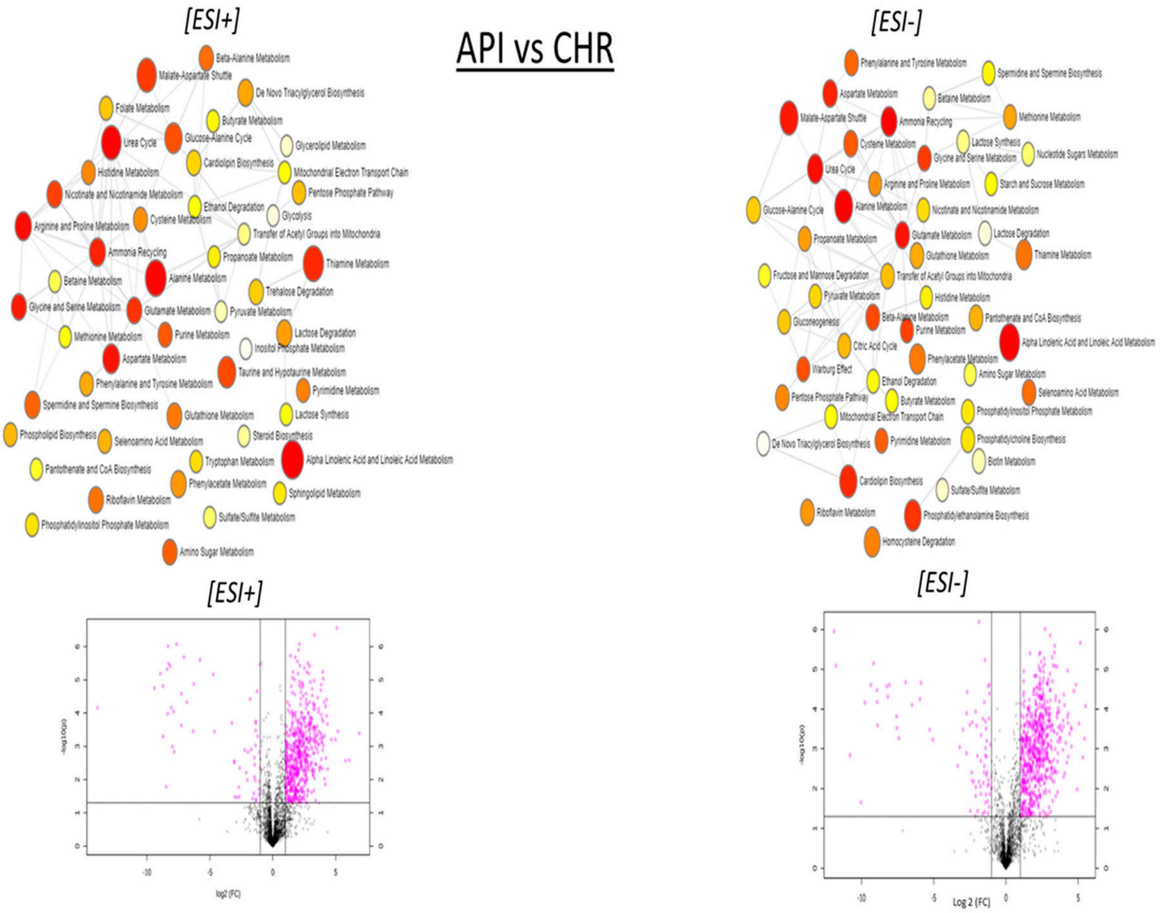

Scheme S5. Metabolites differentially regulated by apigenin and chrysin. Pathway enrichment analysis and volcanic plots provide cluster of metabolites differentially regulated by apigenin and chrysin in positive (left) and negative (right) ion modes.

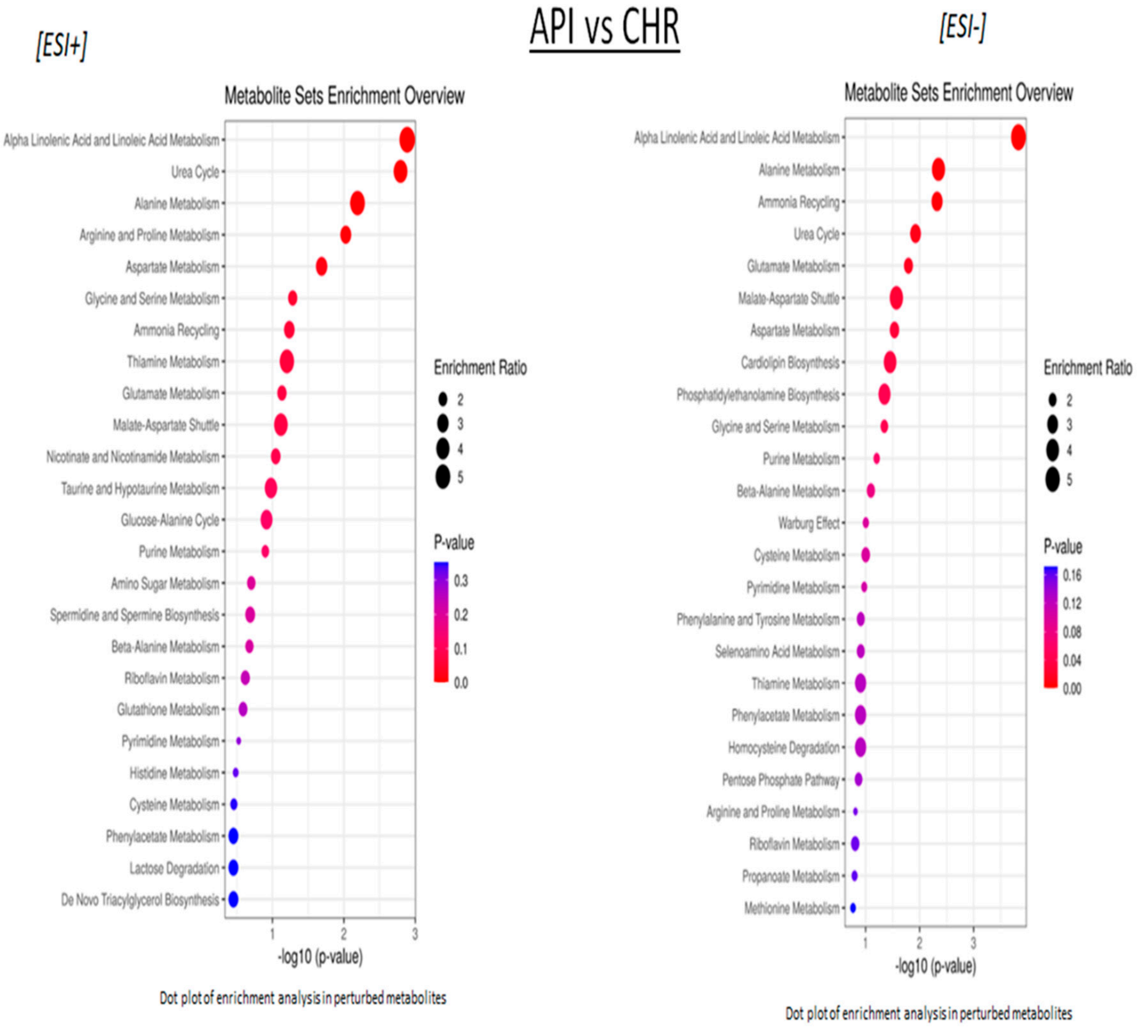

Scheme S6. Dot plot of enrichment analysis of perturbed metabolites in apigenin versus chrysin treated groups. The dot plot of enrichment analysis of metabolites which were altered in apigenin versus chrysin treated groups in positive ion mode (right) and negative ion mode (left) are depicted in this figure. The altered metabolites with the enrichment ratio are depicted in this figure.

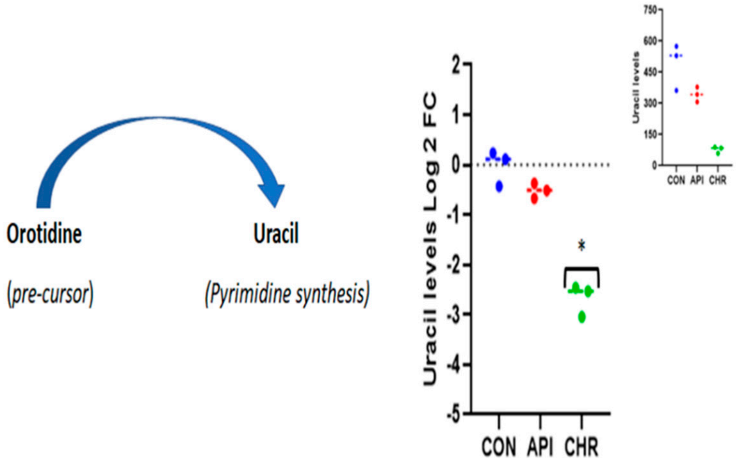

Scheme S7. Decrease in orotidine mediated pyrimidine synthesis by chrysin. Along with orotidine, we also observed a chrysin mediated decrease in uracil synthesis (pyrimidine base). The raw values are presented as upper insert and log 2 fold change values are provided in the figure.

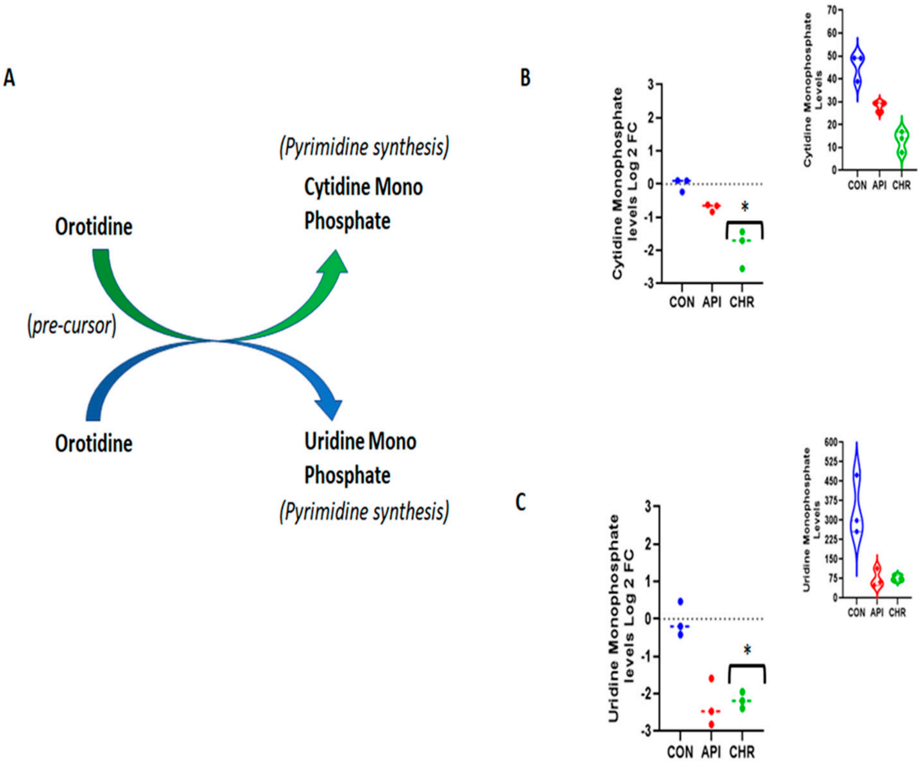

Scheme S8. Decrease in monophosphates of cytidine and uridine with chrysin. A) Orotidine usually gets converted into cytidine and uridine (pyrimidine bases) and the phosphate forms of these nucleotide bases were evaluated from the metabolite panel. The log 2 fold change values and the raw values (upper inserts) for cytidine monophosphate and uridine monophosphate were represented in B) and C) respectively.
